# Emotional Labor: Scale Development and Validation in the Chinese Context

**DOI:** 10.3389/fpsyg.2019.02095

**Published:** 2019-09-18

**Authors:** Chunjiang Yang, Yashuo Chen, Xinyuan Zhao

**Affiliations:** ^1^School of Economics and Management, Yanshan University, Qinhuangdao, China; ^2^Business School, Sun Yat-sen University, Guangzhou, China

**Keywords:** emotional labor, scale development, scale validation, China, surface acting, deep acting, expression of naturally felt emotions, emotion termination

## Abstract

Based on the specific cultural context to add greater theoretical precision to emotional labor, we developed the Chinese version scale of emotional labor. For a comprehensive construct development and validation of the new scale, we carried out five studies. First, we used grounded theory methodologies, performed analysis of data from field observations of 15 frontline employees through insider's angle (Study 1) and in-depth interviews with 35 employees (Study 2), and preliminarily glimpsed the main content of emotional labor in China. Second, combined with existing literature and open-ended questionnaires with 110 employees (Study 3), we identified four dimensions labeled as surface acting, deep acting, expression of naturally felt emotions and emotion termination, and established the initial items. Third, Study 4 with 166 service workers from China was performed to demonstrate the validity, reliability, and acceptable psychometric properties of the scale and thus formed the formal scale. Finally, multi-wave data with 403 Chinese samples (Study 5) were collected for validating the formal scale. Future researchers can employ this validated scale to investigate emotional labor in Chinese service settings. We expect the emotional labor phenomena in the Chinese context can add valuable and novel insight into the stock of emotional labor knowledge in numerous geographical and cultural contexts.

## Introduction

The service sector with important economic importance and social significance has been one of the fastest-growing segments of the Chinese economy. In 2017, the added value of China's service industry has reached 42,703.2 billion Yuan, accounting for 51.6% of the country's GDP (Wang, 2018). Increased competition among service providers, along with overall growth in the service economy, has made delivering superior service become more demanding than ever before (Teng, 2019). Organizations are increasingly willing to depend on service providers' presentation of emotions to the customer in service interactions (Chi and Grandey, 2019). As a consequence, emotional labor, generally defined as the act of expressing organizationally desired emotions during service transactions, is a key component of the work performed by service workers worldwide (Ashforth and Humphrey, 1993; Chu and Murrmann, 2006). Research has shown that positive affective displays, namely “emotional labor” are positively associated with important customer outcomes, such as customer satisfaction, customer loyalty, and intention to return (Hennig-Thurau et al., 2006; Groth et al., 2009). However, those studies root almost in the western context (Grandey et al., 2005, 2019). More and more evidence has indicated that conceptions and associated practices of emotional labor have been found to vary widely across cultures, especially between the East and the West (represented by China and the USA) (Luo et al., 2019). For example, Allen et al. (2014) found that many of the relationships among emotional labor variables vary as a function of the cultural context using samples of U.S. and Chinese service workers. Consistent with this, Grandey et al. (2005, p. 902) have proposed that “cultural differences in work emotions are important to understand.”

Confucius admonished that the mechanic, who wishes to do his work well, must first sharpen his tools (Farh et al., 2006). Similarly, As Korman (1974, p. 194) has said “The point is not that adequate measurement is “nice.” It is necessary, crucial, etc. Without it we have nothing.” Unfortunately, the existing research, which almost is Chinese translations of Western measures, lacks consideration of the local context (Farh et al., 2006; Zhang and Zhu, 2008; Chen et al., 2012). The need for Chinese culture-based valid research instruments of emotional labor takes on even greater urgency. On the one hand, indiscriminate use of the Western emotional labor scale inevitably misses the unique aspects of Chinese emotional labor. On the other hand, the dearth of valid operationalizations has made research findings compromised. Additionally, researchers are calling for the development of instruments to measure emotional labor based on different historical and cultural backgrounds (Nixon et al., 2019). Against this backdrop, the purpose of the current investigation is to systematically develop and validate an emotional labor measure for organizational settings of China.

## Literature Review

### Emotional Labor

Emotional labor, first introduced by Hochschild (1983, p. 7), is defined as “the management of feelings to create a publicly observable facial and bodily display.” Hochschild's (1983) definition of emotional labor implicitly presumes that servicers more or less consciously attempt to manage emotion by engaging in surface acting (employees modify their displays without shaping inner feelings) or deep acting (employees modify internal feelings to be consistent with display rules). Earlier studies indicated that the concept of emotional labor was developed around three themes: internal states, external behavioral displays and internal processes (Hochschild, 1979, 1983; Glomb and Tews, 2004). Ashforth and Humphrey (1993) defined the act of displaying the appropriate emotion (i.e., conforming with a display rule) as emotional labor which both emphasized behavior and decoupled the experience of emotion from the expression of emotion. Meanwhile, they viewed genuine experience and expression of expected emotion (one spontaneously and genuinely experiences and expresses the expected emotion) as a third means of accomplishing emotional labor. Surface acting, deep acting, and genuine experience of emotions involve and emphasize the internal state of emotional harmony, emotional dissonance, or emotional deviance. Then, Glomb and Tews (2004) provided strong evidence for the existence of those three types of emotional labor. Importantly, Diefendorff et al. (2005) found that employees from a wide range of industries expressed genuine emotions in the service process. Expression of naturally felt emotions is probably the most common of the three strategies for emotional labor. Meanwhile, Jordan et al. (2008) also found that many employees were skilled at performing genuine emotional expression, which was the most effective form of emotional labor. Morris and Feldman (1996) suggested that emotional labor be best conceptualized in terms of four distinct dimensions: frequency of appropriate emotional display, attentiveness to required display rules, variety of emotions to be displayed, and emotional dissonance. This theoretical approach focuses on the external behavioral displays of employees (Brotheridge and Lee, 2003). Additionally, some scholars focus on the internal processes involved in creating an emotional display: typically self-regulation processes (Grandey, 2000; Brotheridge and Grandey, 2002). Overall, previous perspectives of emotional labor all have the same underlying theme: individuals can control their emotional expressions at work. Emotional labor, thus, is the process of regulating both feelings and expressions for the organizational goals.

### Emotional Labor Across Cultures

Culture builds and prescribes situational scripts in interpersonal contexts in different ways (Choi H. et al., 2019). Cultural context shapes and influences the emotional expression of people from any culture and dictates what, how, when, and to whom people should express their emotional experiences (Ekman and Friesen, 1969; Allen et al., 2014; Yang et al., 2019). Researchers have studied that emotion regulation and display rules for experiencing emotions have significant differences across cultures (Eid and Diener, 2001; Masuda et al., 2008; Safdar et al., 2009). For instance, Grandey et al. (2005) showed that the way that emotional labor is enacted by service employees differed between the U.S. and France. Specifically, they found that the difference stems from an impulsive orientation of French culture and an institutional orientation of the U.S. culture. In Safdar et al.'s (2009) multinational study, they found a striking difference in emotional display rules among three cultural groups, namely Japan, Canada, and the United States. Allen et al. (2014) found that the emotional regulation strategies (i.e., surface and deep acting) are reported as being used more often in China than in the U.S. Luo et al. (2019) suggested that there should be observable cultural differences of emotional labor in individualistic and collectivistic cultures. In an impressive study using data from 3,570 six- to 15-year-olds from 13 Asian, European, Middle Eastern, and South American countries, Scherr et al. (2019) found that cultural indicators explained the amount of variance in children's expression of the basic emotions of anger, fear, sadness, and happiness. The scholars widely acknowledged that the main distinctions made between Eastern and Western cultures are that they differ in the levels of collectivism-individualism, power distance, and uncertainty avoidance (Hofstede, 1980; van Hemert et al., 2007; Uchida et al., 2008; Kitirattarkarn et al., 2019). Accordingly, differences in emotional labor across cultures can be mainly explained by those cultural factors.

### Emotional Labor and Chinese Culture

Chinese culture, as representatives of a collectivistic culture, values groups rather than individuals, and promotes harmony within the group (Noon and Lewis, 1992; Chi et al., 2019). Emotions are seen as interactive experiences. As a result, emotional moderation and emotion control have priority and the Chinese would be less inclined to express powerful emotions (Safdar et al., 2009). In contrast, individualism embedded in western culture encourages outward displays of emotion, and individuals are more comfortable expressing their natural emotions (Lu and Guy, 2019). In addition, a high level of power distance context makes the Chinese identify the unequal distribution of power within society and encourages self-repression when interacting with people of higher status (Matsumoto, 2007). Generally speaking, servicers are prone to respect the identity of customers and thus they often compromise in conflicts with customers. Uncertainty Avoidance refers to the degree to which people feel threatened by the unknown or ambiguous situations and have developed beliefs, institutions, or rituals to avoid them (Hofstede, 1980). Chinese cultures inclined to this value orientation are associated with greater levels of anxiety and fear responses in unknown or ambiguous situations and result in programmed emotions or even no responses (Adair and Xiong, 2018; Liu and Luo, 2019). Overall, collectivism, high power distance, and high uncertainty avoidance relate directly to emotional expression and management with more emotional control, moderation, and suppression.

To enhance readability, this development process has been separated into four study phases, each addressing a different purpose. First, we gathered related information and examples of emotional labor through field observations of 15 frontline employees from an insider's angle (Study 1) and in-depth interview with 35 employees (Study 2). Second, we confirmed the local content, dimension, and preliminary items of emotional labor by open-ended questionnaires with 110 employees (Study 3) and existing literature. Third, we got the formal scale of Chinese version of emotional labor through survey with 166 service workers from China (Study 4). Fourth, we examined psychometric properties, convergent and discriminant and criterion-related validity of formal scale by multi-wave data with 403 Chinese samples (Study 5). In the following sections, we present the five studies in detail followed by a general discussion.

## Methods

We used grounded theory methodologies, which provided a systematic and rigorous set of procedures and techniques for collecting and analyzing data and allowing a comprehensive understanding of complex issues (Creswell et al., 2007).

## Study 1 Field observation

The researcher conducted a one-month participant observation as a service employee in a three-star hotel. This real insider's angle and rich experience are critical in the qualitative study. This intimacy with the phenomenon of interest means that the reader can see the world through the researcher's eyes, which often captures the informants' experiences (Bansal and Corley, 2011). During the period, the content of observation was 2-fold: (i) the emotional changes, emotion expression, and emotional response to customer requirements of service employees in the process of service interaction; (ii) the frontline employees' emotional change and emotional recovery when the service ends. At the end of each day, the researcher collated, archived and analyzed the notes and auto-recording at the observation site.

### Participants

Among 15 respondents, 2 were males and 13 were females; 5 were married and 10 were single; their ages ranged between 20 and 45 years old (average age = 28) and their education background ranged from junior high school to bachelor's degree. The respondents had 0.5–9 years of work experience. Work positions include service staff, cook, superior, and manager.

### Content Analysis

We used the three-step procedure to analyze the data (Devitt, 2003). First, original materials were filtered and we got 124 valid units. Second, we grouped the 124 units into meaningful and interpretable categories, and a total of 36 subject terms were obtained. Through the content analysis, three semantic relationships existed in 36 subject terms, namely, equivalent relationship (different statements had the same meaning), correlational relationship (different sentences explained and elaborated on the same phenomenon or problem from different perspectives) and incompatible relationship (there was no intersection among contents contained in different subject terms). Specially, 36 subject terms were grouped into eight equivalent relationships, three correlational relationships, and four incompatible relationships. Finally, five subcategories were derived according to 36 subject terms and their relationship categories, namely, factors influencing service attitude, negative service, positive service, express true feelings, and ways to deal with emotions.

### Discussion

We found that workloads, salary, customers' attitudes, leaders' attitudes and conflicts with customers, are the main factors influencing the service emotions. Employees are easily prone to induce negative emotions, such as fidgets, anxious, and angry due to the characteristics of heavy service intensity in the workplace. In the case, an unfriendly attitude of a customer is more likely to inflame conflicts while leaders' help is timely rain. Help from leaders determines employees' service emotions, attitudes, and strategy to a large extent. Also, the emotional management strategies mainly included expressing positive emotions, expressing negative emotions, self-regulating emotions, and expressing real emotions.

## Study 2 In-depth Interviews

To further gather related information and examples of emotional labor in the Chinese context, interviews were carried out in a larger scope than Study 1.

### Participants and Procedure

The samples mainly came from traditional service industries, such as hotels, hospitals, and retailers from China. A total of 35 participants were interviewed, including doctors and salesmen. In terms of age, 90% aged between 20 and 40 years old. The overall education was medium level, with 50% junior college level or below, 40% undergraduate level or above, and 10% others. Forty-five percent were male and 55% were female. Seventy percent of the samples were from frontline positions, and 30% were from management positions. Participants were provided with a written information sheet describing the study and informing them of their right to confidentiality, anonymity, and withdrawal from the study at any time. Data was collected using semi-structured, in-depth interviews. The researcher interviewed each participant face-to-face. The average interview length was 1 h. A contact summary sheet was completed to document all communication with participants. The interviewees were asked to respond to the following open-ended questions: What factors affect your service attitudes at work? What kinds of service approaches will be taken and why? In conflicts with customers, what approaches will be taken to resolve them and why? When the workload is heavy, what are your emotions and what kinds of service strategy will you adopt? During the interviews, comprehensive notes of interviews were recorded carefully.

### Content Analysis

Similarly, interview materials were required to be simplified and we got 177 valid analysis units. Judges A and B compared all the units to determine similarities and differences and perform necessary merging or separation. One hundred seventy-one analysis units have grouped the units into 41 related subject terms with 10 pairs of correlational relationships, 7 pairs of equivalent relationships, and 9 pairs of incompatible relationships. Finally, we found the five subcategories in accord with the result of Study 1.

### Discussion

Through content analysis of all the coded notes, we found that the amount of workload, salary, and conflicts with customers are primarily related to employees' service emotions, attitudes, and behaviors. When the workload was heavy, employees would not take customers seriously but save time to service other waiting customers. However, service workers may apologize to the customer and keep servicing according to the requirements of the customer when they have conflicts with customers. The results of the interview preliminarily suggest that Chinese emotional labor is related to emotional expression, management, and control and it is also affected by the amount of workload, salary, and conflicts with customers.

## Study 3 Open-Ended Questionnaire

The objective of Study 3 was to further confirm the local contents and dimensions of emotional labor in the Chinese Context. Given that the data collection of Study 1 and Study 2 were concentrated in traditional service industries, Study 3 would collect a larger sample by open-ended questionnaires to expand the applicability of native emotional labor. The respondents were mainly front-line employees in the service industry, such as clothing guide, bank teller and driver. The questionnaires were distributed and collected in electronic and paper forms. The questions of the questionnaire were compiled based on the result of Study 1 and 2. Heavy workload and conflict with customers most induced the service employees to appear the emotional fluctuation and to adopt the corresponding emotional management approaches. Therefore, we set heavy workload and service conflicts as the working situation to explore the emotional expression, management, and control of servicers in two situations. The questions were presented as follows: When work is urgent and customers are numerous, what is your psychological state and how would you treat your customers? What is your psychological state when you have a disagreement/conflict with a customer and what would you do?

### Participants and Procedure

One hundred thirty questionnaires were distributed and 110 were recovered. Among respondents, 46% were male, and 89% aged between 20 and 30. In terms of education, about 76% were junior college graduates, 15% were undergraduates and 9% were others. Front-line service employees accounted for 91% of the respondents. In terms of work tenure, the longest one was 25 years, and the shortest one was 1 year, with an average of 5.7 years. Industry distribution covered several industries such as banking and medical and health care.

### Content Analysis

We obtained 1,332 initial statements by conceptualizing the data collected from the open-ended questionnaires. Then, 244 statements unrelated to emotional labor and 43 semantically ambiguous statements were deleted. The remaining 1,045 sentences were performed necessary merging or separation. We identified 211 primary subject terms and further got 22 subcategories. Of the subcategories, 12 subcategories (E_1_-E_12_) were associated with expression, control, or manipulation of emotions during service encounters and were thus believed to tap the emotional labor construct.

### Dimensions of Emotional Labor

#### Surface Acting

Service providers' inner thoughts might be contrary to external behaviors. They usually expressed temporary and surface emotions and seldom revealed their real feelings in the process of interactions with clients.

“When customers were many,” waitress A was waiting for the customer order. But customers were slow to order the menu. Thus, a trace of discontent and impatience crossed her face, and then she tried to conceal, kept a positive attitude, and smiled toward our customers although she was already anxious. Considering that the customer is god, she had to hide her true feelings.”

Our finding was consistent with previous research. For example, Ashforth and Humphrey (1993) believed that surface acting was the mismatch between an individual's inner feelings and the requirements of the organization. In this case, employees only changed the external body language and facial expression to meet the rules of the organization while they did not change their inner feelings. Related information of surface acting including its definition, subcategory, typical statement in interview and frequency are presented in Table 1.

**Table 1:** The summary of dimensions of emotional labor.

**Core category**	**Definition**	**Subcategory**	**Typical statement**	**Frequency**
Surface acting	When the internal feelings of an individual do not match the requirements of the organization, the external body language, and expressions are changed to meet the rules of the organization	E_1_: Egoism	Comfort myself	49
			People are selfish	
			Analyze the problem from one's own point of view	
		E_2_: Economic interest	Work for pay	18
			The rise of salary reduces the emotions	
			Hold out for pay	
		E_3_: Control emotions	The customer is always right	271
			Maintain a good attitude anyway	
			Learn to control emotions	
		E_4_: Obedience	Work according to customers' requirement	66
			Subject to the customers' requirement	
			Tolerate customers' demands	
		E_5_: Ignore customers	Don't care about customers' feedback	62
			No consideration for customers	
			Don't care about the customers' emotions	
Deep acting	Think deeply about the situation and try to change one's own inner feelings to match the required displays of organizations	E_6_: A heart and soul for customers	Analyze the problem from the customer's point of view	143
			Serve from the customer's point of view	
			Put yourself in the customer's shoes	
		E_7_: Serve customers sincerely	Encounter good patient service attitude will improve	154
			Customer affirmation promotes work attitude	
			Customers with a good attitude promote their own service	
Expression of naturally felt emotions	Express one's own true emotions	E_8_: Express positive emotions	If happy, express directly	25
			Express happy directly	
			Express your happiness directly	
		E_9_: Express negative emotions	I'm not willing to suppress my emotions	129
			I get into fights with customers	
			No willing to regulate one's own emotions	
Emotion termination	Stop generating internal feelings and conveying external emotions	E_10_: Silence	Be silent if customers don't listen to explanations	86
			Treat customers coldly	
			Listen to customers' complaints	
		E_11_: Regardless	Let nature take its course	60
			Ignore difficult customers	
			Ignore customers when they are annoying	
		E_12_: Escape	Avoid contact with customers when conflicts arise	63
			No emotional response	

#### Deep Acting

The second dimension of emotional labor is that service workers manipulate or change their inner state and actively express emotions and behaviors.

“I face difficulties positively during work. For example, when I have conflicts with customers, I would carefully explain to them, understand their needs and provide services based on their interests.”

When serving customers, service workers deliver real services to customers based on directly changing their inner feelings. Compared with surface acting, it has the advantage of sincerity. Similarly, prior literature has defined that deep acting is that service workers will spontaneously think deeply about their service role, change or enhance their inner feelings through their efforts, and finally express the emotions that conform to the rules of the organization regardless of their genuine feelings (Morris and Feldman, 1996; Diefendorff et al., 2005). Related information of deep acting including its definition, subcategory, typical statement in interview, and frequency are shown in Table 1.

#### Expression of Naturally Felt Emotions

The service workers do not need to consciously attempt to manage emotions (Grandey, 2000). It implies emotional labor allows one to spontaneously and genuinely experience and express true emotions. In other words, a service agent may naturally feel what he or she is expected to express without having to work up the emotions. Thus, the genuine expression of true emotion serves as a third means of accomplishing emotional labor. In correspondence with that, Humphrey et al. (2015) strongly pointed that researchers should start routinely including natural and genuine emotions in their studies of emotional labor. Moreover, convincing evidences have supported that the expression of naturally felt emotions is very important for highlighting the bright side of emotional labor (e.g., Diefendorff and Richard, 2003; Gabriel et al., 2015). Service providers naturally pass on positive emotions when they are engaged in chatting with customers merrily while they do not hide their negative feelings in case of disagreement with customers.

“No need to hide in front of customers, because when you do, customers could feel it. It's better to be yourself.”

The difference between genuine expression of emotion and surface acting is that this emotional expression strategy is not modified and processed, while surface acting is a kind of cover-up for the individual's inner true feelings. Deep acting is the result of the individual's efforts to adjust inner feelings. Related information of expression of naturally felt emotions including its definition, subcategory, typical statement in interview, and frequency are presented in Table 1.

#### Emotion Termination

Through sorting and analyzing sources, we also found that Chinese service workers would also display other kinds of accomplishing emotional labor which is different from the above three types of emotional expression strategy.

“When they encounter problems, most of them will explain to customers to get forgiveness. But when customers don't listen to their explanation, they will feel frustrated and treat customers in silence, such as quietly listening to customers' complaint or indifferent to customers.” “Some workers just let nature take its course when faced with problems and keep continuing to work in accordance with organizations' rules and regulations, regardless of customer comments, and requests.” “Some workers also choose to escape from problems, such as complete lack of emotional response to customers.”

The common characteristic of the above expression strategies is that there is no further inner emotional fluctuation and no emotional transmission to the outside. As the name implies, emotion termination refers to the idea that service workers take efforts to stop conveying their internal and external emotions, especially when it comes to conflicts with customers. Each conceptualization of emotional labor assumes that emotions are being managed at work to meet the display rules stated by the organization. Firstly, emotion termination needs great effort to finish because negative feelings are normal when coping with difficult customers. Secondly, although it is against the required displays of organizations, emotion termination seems to be a smart way for most service employees to achieve organizational goals, especially in complex and uncertain situations. The significant difference from surface acting is that surface acting conforms to the organization's expression rules. Surface acting is internally false, and represses inner feelings. In emotion termination, employees consciously modify their displays by no emotional displays and inner feelings. Related information of emotion termination including its definition, subcategory, typical statement in interview, and frequency are presented in Table 1.

Surface acting, deep acting, expression of naturally felt emotions and emotion termination do comprehensively cover all the ways one may manage emotions at work. In short, we suggest that the four proposed dimensions do completely define the emotion management of Chinese employees.

#### Data Secondary Coding

To ensure content validity and accuracy, two organizational behavior experts who are familiar with grounded theory worded the items of the open-ended questionnaires, compared the lists for similarities and differences, combined, revised, or deleted the items once again. After several rounds, both experts agreed on the dimensions proposed by the author.

#### Item Generation

In an attempt to represent comprehensively workplace experiences of emotional laborers and thus increase content validity, anecdotes, and descriptions were obtained from the general body of emotional labor literature and semi-structured interviews and open-ended questionnaires. First, Diefendorff et al. (2005) provided a three-dimensional structure including deep acting, surface acting, and the expression of naturally felt emotions, which was consistent with the three dimensions of Chinese emotional labor. Second, Diefendorff et al.'s (2005) scale was widely used worldwide with good reliability and validity (Judge et al., 2009). Specifically, many domestic scholars (including scholars in Taiwan) chose Diefendorff et al.'s (2005) scale to conduct empirical research on domestic employees' emotional labor, which proved the applicability of the scale in the Chinese context. Thus, translation and back-translation of Diefendorff et al.'s (2005) scale were conducted in order to finish the measure of surface acting, deep acting and genuine expression of emotion. In addition, we selected four items of each dimension from high to low according to their load coefficients in the original scale for following the principle of accurate measurement and convenient application. The items of these dimensions are listed in Table 2.

**Table 2:** All items of Chinese emotional labor.

**Dimensions**	**Items**	**Chinese translation**	**Application examples/frequency**
Surface acting	A_1_: I put on an act in order to deal with customers in an appropriate way	工作中为了让顾客满意，我不得 不掩饰真实的自己	Grandey (2003) Cossette and Hess (2015) Mishra et al. (2012)
	A_2_: I just pretend to have the emotions I need to display for my job	工作中工表达的服务态度，通常 不是我的真实感受。	Grandey (2003) Cossette and Hess (2015) Mishra et al. (2012)
	A_3_: I put on a “mask” in order to display the emotions I need for the job	为了表现好的服务态度，我经常 隐藏真实感受。	Grandey (2003) Cossette and Hess (2015) Mishra et al. (2012)
	A_4_: I fake the emotions that I must show to customers	面对顾客时，我表达的情绪往往 不是真实的。	Kruml and Geddes (2000) Cossette and Hess (2015) Mishra et al. (2012)
Deep acting	B_1_: I try to actually experience the emotions that I must show to customers	为了表现**出**好的服务态度，我时 常试着去体验顾客的感受。	Grandey (2003) Cossette and Hess (2015)
	B_2_: I make an effort to actually feel the emotions that I need to display toward others	通常情**况**下，为了向顾客传递良 好的情绪我可以改变自己的态 度。	Grandey (2003) Cossette and Hess (2015)
	B_3_: I work hard to feel the emotions that I need to show to customers	向顾客表现**出**的态度，是我对情 绪控制后的结果。	Grandey (2003) Cossette and Hess (2015)
	B_4_: I work at developing the feelings inside of me that I need to show to customers	通过自身的调整和控制，我展示 给顾客的情绪是真实的。	Kruml and Geddes (2000) Cossette and Hess (2015)
Expression of naturally felt emotions	C_1_: The emotions I express to customers are genuine	我向顾客表达的情绪是真实的。	Diefendorff et al. (2005) Cossette and Hess (2015)
	C_2_: The emotions I show customers come naturally	我向顾客表达的情绪是没有经过 掩饰的。	Diefendorff et al. (2005) Cossette and Hess (2015)
	C_3_: The emotions I show customers match what I spontaneously feel	我向顾客表达的情绪是自然流露 **出**的。	Kruml and Geddes (2000) Cossette and Hess (2015)
Emotion termination	D_1_: When there is disagreement with the customer, I will serve according to the customer's requirements without any emotional change	当和顾客意见不一致时，我按照 顾客的要求服务，不会有情绪变 化。	151
	D_2_: When customers disapprove of my service, I will choose silence	当顾客不认可我的服务时，我会 选择沉默。	189
	D_3_: I have no feelings when customers' demand is too much or difficult to satisfy	当顾客要求过多或一时难以满足 时，我没有任何感觉。	193
	D_4_: When customers ask for too much, I just work by the organizations' rules without emotions	当顾客要求过多时，我不带任何 情绪且只按照**公**司制度工作。	247
	D_5_: When I cannot communicate with customers normally, I will choose not to communicate and without any emotions	和顾客不能正常沟通时，我会选 择不沟通且没有任何情绪表现。	253

The emotion termination is a distinct strategy for managing emotions at work and is included in emotional labor in the Chinese context. We selected five items with the highest frequency in the statistical results of the open-ended questionnaire survey. Detailed items of emotion termination are listed in Table 2.

## Study 4 The Formal Scale

The above items of Chinese emotional labor were incorporated into a questionnaire for a pilot study. The purpose of this process was to confirm expectations regarding the psychometric properties of the new measure (Hinkin, 1995).

### Participants and Procedure

We asked respondents to evaluate, using a seven-point Likert-type scale (“1” = “strongly disagree,” “7” = “strongly agree”). The pre-survey was conducted in five typical service organizations, including hotels, shopping malls, banks, telecom companies, and port logistics companies. The survey was mainly conducted on the front-line service personnel of the above enterprises (*N* = 166; 55% male; 71% = between the age of 20–30 years; 32.4% = bachelor's degrees or above; 80% = frontline employees). Churchill (1979) suggested that the purification of a measurement instrument should begin with the computation of the coefficient α. The coefficient α of each dimension can be seen in Table 3.

**Table 3:** Coefficient α and exploratory factor analysis results of the Chinese emotional labor.

**Dimensions**	**Cronbach'sα**	**Items**	**Cronbach'sα after deleting the item**	**Factor extraction**	**Factor loading**	**% variance explained**
Surface acting	0.755	A_1_	0.730	Factor1	0.452	29.208
		A_2_	0.706	Factor 1	0.606	
		A_3_	0.642	Factor 1	0.339	
		A_4_	0.710	Factor 1	0.211	
Deep acting	0.306	B_1_	0.019	Factor 2	0.728	14.594
		B_2_	0.090	Factor 2	0.831	
		B_3_	0.476	Factor 3	0.347	
		B_4_	0.381	Factor 4	0.807	
Expression of naturally felt emotions	0.843	C_1_	0.749	Factor 4	0.854	9.425
		C_2_	0.856	Factor 4	0.754	
		C_3_	0.726	Factor 4	0.859	
Emotion termination	0.565	D_1_	0.548	Factor 3	0.721	8.750
		D_2_	0.360	Factor 3	0.831	
		D_3_	0.412	Factor 3	0.694	
		D_4_	0.594	Factor 1	0.792	
		D_5_	0.569	Factor 2	0.511	

The Kaiser–Meyer–Olkin (KMO) measure and Bartlett's test of sphericity were used to ensure that the data had inherent sufficient correlations to perform exploratory factor analysis (EFA). The KMO index was 0.763, and Bartlett's test of sphericity was significant at a level of 0.00, which justified the use of EFA. An EFA was performed on the emotional labor items. The extraction method for EFA is principal component analysis on the premise of eigenvalue >1 and maximum variance skew rotation method. A total of four component factors were extracted, which explained 61.977% of the total variance. Table 3 shows items, EFA factor loadings, and percentage of variance explained.

### Discussion

The value of the coefficient α ranged from 0.306 to 0.843 for the four dimensions and this implied that it was necessary to remove some items from each dimension to improve the α value. The standardized loadings of items A_1_, A_3_, A_4_, and B_3_ were <0.5, indicating that the convergence effect of item A_1_, A_3_, A_4_, and B_3_ are not good, while other items are acceptable. Item B_3_ and item B_4_ were the measurement questions of deep acting, but they were classified as other dimensions of emotional labor in EFA. The same problem appears in EFA for items D_4_ and D_5._ The two items are classified into two categories of factors, respectively. Given the above problems, we carried out a detailed analysis of the reasons and proposed solutions.

First, item B_3_ and item B_4_ belong to the translation version, and there are inevitable errors, misunderstandings, and incomprehension in the translation process. We hope to solve the problems with a second translation carefully. Second, items D_4_ and D_5_ are independently compiled in the Chinese context, and they perhaps overlap with other items of emotional labor rooted in western countries because of the essence of emotional labor. Based on the results of the preliminary survey, we further modified these items to measure the connotation of emotion termination more accurately and appropriately. According to the results of scale revision, the formal scale is in Table 4.

**Table 4:** The formal scale of Chinese emotional labor.

**Dimensions**	**Items**	**Factor loading**
Surface acting	I put on an act in order to deal with customers in an appropriate way	0.804
	I just pretend to have the emotions I need to display for my job	0.895
	I put on a “mask” in order to display the emotions I need for the job	0.815
Deep acting	I try to actually experience the emotions that I must show to customers	0.675
	I make an effort to actually feel the emotions that I need to display toward others	0.789
	In order to satisfy customers, I will try to understand their feelings	0.823
Expression of Naturally felt emotions	The emotions I express to customers are genuine	0.759
	The emotions I show customers come naturally	0.868
	The emotions I show customers match what I spontaneously feel	0.757
Emotion termination	When there is disagreement with the customer, I will serve according to the customer's requirements without any emotional change	0.638
	When customers disapprove of my service, I will choose silence	0.787
	I feel helpless when customers ask too much or are unable to meet them temporarily	0.775

## Study 5 The Formal Scale Validation

The main goal of Study 5 was to test the validity of the formal scale from psychometric properties, convergent and discriminant, and criterion-related validity. This provides additional validity for the construct measures developed in Study 4. Specific objectives were to (a) increase reliabilities of the Chinese emotional labor scale and (b) contribute to the evidence of construct validity by testing hypotheses regarding emotive labor, burnout, and job satisfaction.

### Emotional Labor Outcomes

The bulk of the previous research on emotional labor has focused on the potential negative consequences of emotional labor for the psychological well-being of employees (Pugliesi, 1999; Yanchus et al., 2010; Yin et al., 2019). We consider the impact of the four dimensions of emotional labor on the psychological well-being most frequently investigated in emotional labor research, namely, burnout, and job satisfaction.

#### Burnout

Burnout is a severe psychological and physical response syndrome resulting from prolonged stress and frustration at work, which occurs frequently among individuals who are emotion labors (Maslach et al., 1986). It is a multidimensional concept with three components: emotional exhaustion, depersonalization, and reduced personal accomplishment (Maslach et al., 2001). Emotional exhaustion involves constant emotional overextension and being drained through contact with other people. With depersonalization, employees take a dehumanizing and impersonal view of others, treating them callously (Cheng and Yang, 2018). Reduced personal accomplishment refers to a loss of or a decline in feeling competent and successful at work.

#### Surface Acting and Burnout

In surface acting, one modifies the displays without shaping the inner feelings which could result in the depletion of personal resources (Trougakos et al., 2015). They experience emotional dissonance, or stress when expressions and feelings diverge (Hochschild, 1983). In addition, surface acting threatens the individual's sense of self (Pugh et al., 2011). Erickson and Wharton underscored that “attempts to control the emotions of workers reach into the very heart of an individual's sense of self” (Erickson and Wharton, 1997 p. 192). The inauthenticity of this surface-level process may result in feeling detached not only from one's true feelings but also from other people's feelings over time, suggesting a relationship with depersonalization. Feeling diminished personal accomplishment is also likely if the employee believes that the displays are not efficacious or are met with annoyance by customers. Thus, surface acting is expected to relate to all three dimensions of burnout. Chen et al. (2019) found that surface labor leads to more burnout.

**H1:** Surface acting will be positively related to emotional exhaustion and depersonalization, and negatively related to personal accomplishment.

#### Deep Acting and Burnout

Deep acting is the process of controlling internal thoughts and feelings to meet the mandated display rules. Deep acting minimizes emotional dissonance by bringing feelings in line with expressions. Thus, deep acting may have a weaker negative relationship with emotional exhaustion (Humphrey et al., 2015; Choi H.-M. et al., 2019). Hochschild (1983) argued that doing “emotion work” was a way of decreasing a state of emotional dissonance and may also result in a feeling of accomplishment if the performance is effective. Brotheridge and Lee (2003) found that deep acting had a positive correlation with a sense of personal accomplishment. Deep acting also involves treating the customer as someone deserving authentic expression. We expected deep acting to relate to lower depersonalization.

**H2:** Deep acting will be negatively related to emotional exhaustion and depersonalization, and positively related to personal accomplishment.

#### Expression of Naturally Felt Emotions and Burnout

Emotional labor allows one to spontaneously and genuinely experience and express the expected emotion. Spontaneous and genuine emotions do not need to be monitored and thus are without resource consuming (Humphrey et al., 2015). Expression of naturally felt emotions means absolute freedom which may facilitate self-expression. It enables the service workers to project “authentic self” into the service, that is, a sense of who one is, a sense of what one values and wants, and a sense of how one is connected to others. Real expression of feelings and emotions reflect one's identity which increases one's feeling of competence and achievement (Humphrey et al., 2015). Similarly, it seems likely that the personalization of role enactment afforded by expressive latitude would promote well-being, reduce emotional exhaustion, and depersonalization.

**H3:** Expression of naturally felt emotions will be negatively related to emotional exhaustion and depersonalization, and positively related to personal accomplishment.

#### Emotion Termination and Burnout

As a new dimension of emotional labor, emotion termination is first proposed in our research, and thus there is no related empirical research in literature revealing and supporting its relationship with burnout. Emotion termination implies service providers do not convey any feelings or emotions to their customers. As prior literature has argued, all acts of self-control are effortful, require inhibition, and draw on limited resources (Johnson et al., 2018). When conditions trigger expression's needs and impulses, individuals must expend resources to suppress and inhibit emotions. Engaging in emotion termination, therefore, leads to emotional exhaustion. Through the above observation and interview, we found that employees who adopt emotion termination are often in a helpless and overwhelming situation. In short, we believe that emotion termination is negatively related to depersonalization. In addition, the strategy of emotion termination makes service workers end the emotional interaction with the customer, which accompanies with unsatisfactory service outcomes. There was a significant gap between reality and expectation and service workers' sense of achievement was reduced in the workplace.

**H4:** Emotion termination will be positively related to emotional exhaustion and depersonalization, and negatively related to personal accomplishment.

#### Surface Acting and Job Satisfaction

Lawler (1973) suggested that it is the discrepancy between the employee's perceptions of conditions that should exist and those that do exist that determine job satisfaction. Employees who perform surface acting choose outward displays consistent with display rules but hide or mask felt emotions. Employees who repeatedly suppress their true emotions or fake them to follow the display rules suffer a continuing discrepancy between inner feelings and outward expressions (Grandey, 2000). This emotional discrepancy leads to emotional discomfort and job stress, which in turn causes job dissatisfaction (Zapf, 2002). Previous studies reveal that repeated contact with people expressing fake emotions could increase feelings of inauthenticity, stress, frustration, or conflict and can be an important element in an employee's dissatisfaction with the job (Hochschild, 1983). Bakker and Heuven (2006) found that when employees choose to feign emotion, it is more likely to trigger lowered job satisfaction. Zhang and Zhu's (2008) and Kinman et al.'s (2011) studies found that surface acting was negatively associated with job satisfaction. Thus, the following hypothesis is proposed:

**H5:** Surface acting will be negatively related to job satisfaction.

#### Deep Acting and Job Satisfaction

Employees who perform deep acting attempt to modify internal feelings about display rules or customer contact situations (Hochschild, 1983). When employees make an effort to feel the required emotions, they feel emotional congruence between true feelings and emotional displays, increasing their job satisfaction (Adelmann, 1995). Brotheridge and Grandey (2002) argued that deep acting could bring positive feedback from a customer which increases job satisfaction. Furthermore, deep acting that reduces uncertainty or helps to avoid embarrassing interpersonal situations actually may be associated with increased job satisfaction (Goffman, 1959; Ashforth and Humphrey, 1993; Wen et al., 2019). Yin (2012) and Zhang and Zhu (2008) supported a positive association between deep acting and job satisfaction.

**H6:** Deep acting will be positively related to job satisfaction.

#### Expression of Naturally Felt Emotions and Job Satisfaction

The expression of naturally felt emotion refers to spontaneously experiencing and displaying the felt emotion in the workplace. In other words, employees use their genuine emotions when they interact with their clients. Rutter and Fielding (1988), for instance, found that a perceived need to suppress genuinely felt emotion in the workplace is negatively associated with job satisfaction. Studies on Chinese human service professionals also found that the expression of naturally felt emotion at work was associated with a good quality of work-life (Cheung and Tang, 2009), job satisfaction, and low psychological distress (Cheung and Tang, 2010). Many scholars have reached the consensus of a positive association between the expression of naturally felt emotion and job satisfaction (e.g., Zhang and Zhu, 2008; Cheung et al., 2011).

**H7:** Expression of naturally felt emotions will be positively related to job satisfaction.

#### Emotion Termination and Job Satisfaction

Emotion termination is a strategy of accomplishing emotional labor under compulsion. “Customers do not understand me, and I cannot do anything about it” The phenomenon accounts for a large proportion of qualitative data. The employees who adopt the emotional termination strategy are in an embarrassing situation. Their feelings are suppressed and they try to stop feeling angry and resentful at an annoying passenger. Accordingly, they are not satisfied with their work spontaneously.

**H8:** Emotion termination will be negatively related to job satisfaction.

### Participants and Procedure

The survey used the time-lagged design to ensure the constructs we concerned would be less influenced by common method bias. The data were collected at two-time points with a one-month interval. At Time-1, measures of the Chinese version of emotional labor and demographic information were completed by participants. At Time-2, the measure of burnout and job satisfaction was provided. Measures of burnout and job satisfaction were translated from English to Chinese, using the translation-back translation procedure (Brislin, 1980). Data were collected from three companies of various types (including bank, port office, and hospital) and geographic locations (China's northeast and north area). Of the 431 surveys distributed, 357 were returned for a response rate of 82.8%. Of the 403 valid respondents, 43% were women, 74% were under 30 years old. The average length of work tenure is 4.15 years. Forty-two percent have bachelor's degrees or above.

### Measures

#### Burnout

Maslach et al.'s (1986) measure of burnout was used in this study. This scale consists of 22 items assessing the three dimensions of burnout, including emotional exhaustion, depersonalization, and personal accomplishment. Participants rated each item using a 5-point Likert scale (5 = “Strongly Agree”; 1 = “Strongly Disagree”).

#### Job Satisfaction

Job satisfaction was assessed with three items adapted by Cammann et al. (1979). Individuals responded to each item on a 7-point Likert scale (7 = “Strongly Agree”; 1 = “Strongly Disagree”).

### Results and Discussion

Cronbach's α again was used in the reliability analysis. The Cronbach's α of surface acting, deep acting, expression of naturally felt emotions and emotion termination were 0.714, 0.743, 0.846, 0.758, respectively. The scale reliability of burnout was 0.724. The scale reliability of job satisfaction was 0.731. All scales' reliabilities were good. CFA with maximum likelihood estimation in LISREL 8.3 was utilized to examine the four-factor structure of our scale items by conducting a series of CFAs. CFAs supported our hypothesized four-factor structure (χ^2^ = 85.74, df = 46, RMSEA = 0.061, GFI = 0.94, AGFI = 0.90, NNFI = 0.94, IFI = 0.96, CFI = 0.96, SRMR = 0.060). All items loaded onto the intended factor at a significant level (*p* < 0.001), with factor loadings ranging from 0.53 to 0.76. The emotional labor scale had a good model fit. Other alternative models were specified by combining constructs (Table 5). The results of the model comparison showed that the baseline four-factor model had a significantly better model fit than the alternative models. In summary, these results supported the original four-factor model.

**Table 5:** Comparison of measurement models.

**Model**	**Description**	**χ^2^**	**df**	****χ^2^**/df**	**RMSEA**	**GFI**	**AGFI**	**NNFI**	**IFI**	**CFI**	**SRMR**
M_1_	Null	1113.40	66	16.87	0.23	0.61	0.54	0.22	0.22	0.22	0.22
M_2_	S+N+P+D	718.74	54	13.31	0.21	0.71	0.58	0.41	0.52	0.71	0.16
M_3_	S+D+N,P	610.18	53	11.51	0.19	0.74	0.62	0.46	0.58	0.74	0.16
M_4_	S+N+P,D	561.76	53	10.60	0.18	0.76	0.64	0.54	0.64	0.63	0.16
M_5_	S+D+P,N	417.84	53	7.88	0.15	0.81	0.72	0.62	0.70	0.70	0.14
M_6_	D+N+P,S	532.80	53	10.05	0.18	0.77	0.66	0.67	0.66	0.65	0.14
M_7_	S+N,D,P	415.00	51	8.12	0.16	0.81	0.71	0.63	0.71	0.71	0.14
M_8_	S+P,N,D	245.75	51	4.82	0.12	0.87	0.81	0.77	0.78	0.82	0.12
M_9_	D+N,S,P	417.04	51	8.18	0.16	0.81	0.71	0.62	0.71	0.70	0.14
M_10_	D+P,S,N	234.31	51	4.59	0.11	0.88	0.82	0.80	0.85	0.84	0.10
M_11_	P+N,S,D	161.56	51	3.17	0.086	0.92	0.87	0.87	0.90	0.90	0.076
M_12_	P,N,S,D	85.74	46	1.86	0.061	0.94	0.90	0.94	0.96	0.96	0.060

*S, surface acting; D, deep acting; N, expression of naturally felt emotions; P, emotion termination*.

To establish discriminant validity, we examined the correlations among the four subscales. Surface acting correlates positively with deep acting (γ = 0.262, *p* < 0.05). Surface acting correlates negatively with expression of naturally felt emotions (γ = −0.189, *p* < 0.05). Although surface acting correlates positively with emotion termination (γ = 0.159, *ns*), their relationship was not significant. Deep acting correlates positively with expression of naturally felt emotions (γ = 0.158, *p* < 0.05) and emotion termination (γ = 0.189, *p* < 0.01). Expression of naturally felt emotions correlates positively with emotion termination (γ = 0.241, *p* < 0.01). The square root of the average variance extracted (AVE) for surface acting, deep acting, expression of naturally felt emotions, and emotion termination were 0.694, 0.705, 0.720, and 0.568, respectively. The square root of the average variance extracted (AVE) for every reflective component above 0.5 offers satisfying evidence of discriminant validity because they were larger than all the bivariate correlation coefficients among the components. Thus, it was concluded that all constructs possessed discriminant validity.

### Criterion-Related Validity

We established the criterion-related validity of the emotional labor scale by demonstrating relationships with burnout and job satisfaction. Table 6 presents the descriptive statistics, reliabilities, and correlations for the studied variables. As predicted in H1, surface acting was positively related to emotional exhaustion (γ = 0.137, *p* < 0.01), depersonalization (γ = 0.031, *ns*), and personal accomplishment (γ = 0.119, *ns*). As predicted in H2, deep acting was negatively related to emotional exhaustion (γ = −0.098, *ns*), depersonalization (γ = −0.214, *p* < 0.01), and personal accomplishment (γ = −0.216, *p* < 0.01). As predicted in H3, expression of naturally felt emotions was negatively related to depersonalization (γ = −0.158, *p* < 0.01). As predicted in H4, emotion termination was positive related to emotional exhaustion (γ = 0.110, *ns*) and personal accomplishment (γ = 0.085, *ns*). As predicted in H5 and H8, surface acting and emotion termination were negatively related to job satisfaction (γ = −0.017, *ns*; γ = −0.332, *p* < 0.01). As predicted in H6 and H7, both deep acting and expression of naturally felt emotions were positively related to job satisfaction (γ = 0.266, *p* < 0.01; γ = 0.241, *p* < 0.01). Overall, most evidence supported the nomological network and criterion-related validity of the emotional labor scale.

**Table 6:** Means, standard deviations, and correlations of the study variables.

	**M**	**SD**	**1**	**2**	**3**	**4**	**5**	**6**	**7**	**8**
1.Surface acting	4.390	1.342	**0.694**							
2.Deep acting	5.276	1.148	0.262^*^	**0.705**						
3.Expression of naturally Felt emotions	4.486	1.351	−0.189^*^	0.158^*^	**0.720**					
4.Emotion termination	4.505	1.123	0.159	0.189^**^	0.241^**^	**0.568**				
5.Job satisfaction	4.946	1.081	−0.017	0.266^**^	0.241^**^	−0.332^**^	**0.679**			
6.Emotional exhaustion	3.077	0.769	0.137^**^	−0.098	0.067	0.110	−0.174^**^	**0.762**		
7.Depersonalization	3.024	0.761	0.031	−0.214^**^	−0.158^**^	−0.191^**^	0.077	0.277^**^	**0.681**	
8.Personal accomplishment	2.865	0.927	0.119	−0.216^**^	0.126	0.085	−0.121^*^	−0.032	0.096	**0.741**

*Reliability coefficients for multi-item scales are shown in parentheses on the diagonal*.

The square root of the average variance extracted (AVE) are shown in bold on the diagonal;

** P < 0.01;

* *P < 0.05*.

### Summary

The overall purpose of this study was to develop and examine the scale of Chinese emotional labor which designed to assess the emotional labor in the Chinese context. The development and validity process of Chinese emotional labor was presented in detail. Five studies were conducted to establish and confirm the scale's contents, dimensions, reliability, and validity. The first study was conducted to gather related information and examples of emotional labor in the Chinese context through an insider's identity. The results of the first study got 124 valid analysis units, 36 subject terms and 5 subcategories involving emotional labor. To further gather information for examining emotional labor and provide additional evidence for the results of Study 1, we conducted a second study with an in-depth interview from 35 service providers. One hundred seventy-seven valid analysis units, 41 subject terms and five subcategories in accord with the result of Study 1 were got. For further confirming the local contents, dimensions, and items of emotional labor in a larger sample range, Study 3 used the open-ended questionnaire which was compiled based on the research results in Study 1 and Study 2. The results found and identified four dimensions of emotional labor in the Chinese context, surface acting, deep acting, expression of naturally felt emotions and emotion termination. The main contents of emotional labor in the Chinese context were also introduced in detail. Accordingly, 16 preliminary items were produced on the basic of the previous literature of emotional labor and the findings of the open-ended questionnaires. To further examine the scale property, we conducted Study 4 with data collected from 166 service workers. Reliability analysis, convergent validity, discriminant validity, and EFAs were employed to examine the preliminary scale. The results led to a deletion of four items, rewording individual items and the unidimensionality of each subscale was confirmed for the remaining 12 items. Finally, we conducted the fifth study to confirm psychometric properties convergent and discriminant validity and criterion-related validity using confirmatory factor analysis (CFA) on data collected from 342 Chinese employees.

## General Discussion

In keeping with the tremendous expansion of the service economy in China, cross-cultural researchers have begun to devote increasing effort to the examination of the construct of emotional labor in the Chinese context, with a unique culture different from western culture. Our central contribution is developing the Chinese version of emotional labor designed to measure the emotional labor that Chinese employees present for their clients. Specifically, our findings support a four-dimensional view of the emotional labor construct in the Chinese context. Results indicate that surface acting, deep acting, expression of naturally felt emotions, and emotion termination are four related but separate dimensions of emotional labor. This unique conceptualization of the emotional labor construct, based on Chinese cultural perspective, not only proves helpful in accurately understanding the nature of Chinese emotional labor but also should provide insights regarding emotional labor in other cultural contexts.

In addition, our findings demonstrate that workload, salary, clients' attitude, leaders' attitude, and conflicts with clients are closely related to service performance and emotions in the service process. When service workers generate a buildup of negative emotions, they need to seek ways to vent their emotions, mainly including pouring out to or turning to their family, colleagues, and leaders, as well as self-regulation, going shopping or watching movies. Although the code of organization governing their interactions with clients requires service workers to have positive attitude to their customers, they also express negative service attitude and even ignore customers deliberately when they face an annoying customer or they are in an upset situation.

Finally, this research is the use of a combination of qualitative and quantitative methodology, which constitutes a methodological contribution. Our data offer some advantages that enrich our contributions. The use of qualitative methodologies is especially important because this method allows us to add richness and depth to the study of organizational phenomena unencumbered by what is in the literature. Many of the stories we heard and observed, from more good stories of positively servicing customers to more sensitive stories about ignoring customers, being disgusted with customers, and stopping service, may not have been revealed by informants without the time and effort we invested. In addition, by using quantitative methodologies, we evaluated and verified emotional labor scale's psychometric properties, convergent and discriminant validity and criterion-related validity.

### Research Application

In a highly emotion-charged environment, employees' emotional expression is significant in determining customers' perception of service quality. Therefore, it is important for employers or managers to monitor the emotional labor performed by their employees. The Chinese version of the emotional labor scale presented here is a concise multiple-item scale, exhibiting good reliability and validity, which China's organizations can use to better understand the emotional labor that their employees perform and their clients perceive. The scale could be used on extensive populations in the service industry including workers who deal mostly with internal customers (e.g., managers) and professionals whose emotional displays are not directly supervised (e.g., doctors). The emotional labor rooted in Chinese culture is most valuable and accurate when it is used periodically to track Chinese workers' emotional labor performance, and when it is used in conjunction with an organization's performance assessment practices. Specifically, service organizations could learn a great deal about their employees' performance by measuring how they enact emotional labor and then determining what needs to be done to improve it. In addition, managers can use the scale as a selection tool to identify the “right” employees with strong potential to perform excellent emotional labor. The ability to identify a job candidate's tendency to use a particular type of acting skill to perform emotional labor can help the company to select employees who are genuinely willing to engage in the emotive effort. From a customer's perspective, the scale can provide additional information when it is used in conjunction with customers' feedback. This scale can assess how customers perceive the quality of interaction they have with servers, as well as the emotional presentation that the servers perform. Soliciting such information can help the hospitality organization to better identify the link between emotional labor and customer perceptions to pinpoint the importance of employees' emotional labor performance.

### Limitations

Although this study makes important contributions to emotional labor scholarship, it is necessary to note some potential limitations. First, data were self-report, which could lead to common method variance (Podsakoff and Organ, 1986). Second, although the scale has been tested on multiple samples and achieved good reliability and validity, replication is necessary to further establish the validity of this emerging measure of emotional labor for Chinses service workers. Third, we only provide limited results regarding consequences in the emotional labor model. Future research should continue to explore other variables that may explain more of the variance in emotional labor and emotional labor's consequences.

### Conclusion

To a greater or lesser extent, every job requires a certain degree of emotional labor. In fact, it would be difficult to identify any job that does not require the exercise of emotional labor. An important implication of the social nature of customer–employee interaction is that culture plays a predominant role in influencing how emotional labor is presented by employees to their customers. The concept of appropriate emotional labor in one culture is not always transferable to another. Surface acting, deep acting, expression of naturally felt emotions and emotion termination constitute the content of the Chinese version of emotional labor. Emotion termination as a dimension of emotional labor in Chinese culture was a unique emotional labor strategy used by Chinese service workers. The Chinese version of emotional labor has a variety of potential applications. It can be used by a wide range of China's organizations in assessing employees' emotional labor levels. It can also help in pinpointing areas requiring more managerial attention and action to improve the emotional labor Chinese employees perform. It is our hope that the availability of this instrument will stimulate much needed empirical emotional labor research based on the Chinese context in order to advance the development of Chinese management literature and global emotional labor knowledge.

## Data Availability

The datasets generated for this study are available on request to the corresponding author.

## Ethics Statement

The studies involving human participants were reviewed and approved by School of Economics and Management's ethics committee. The patients/participants provided their written informed consent to participate in this study. Written informed consent was obtained from the individual(s) for the publication of any potentially identifiable images or data included in this article.

## Author Contributions

In preparing for this manuscript, CY generated ideas, did literature review, and drafted the manuscript. YC participated in the discussions and helped to draft the manuscript. XZ helped to update the literature and actively involved in the revising of the manuscript.

### Conflict of Interest Statement

The authors declare that the research was conducted in the absence of any commercial or financial relationships that could be construed as a potential conflict of interest.

## References

[B1] Adair W. L. Xiong T. X. (2018). How Chinese and caucasian canadians conceptualize creativity: the mediating role of uncertainty avoidance. J. Cross Cult. Psychol. 49, 223–238. 10.1177/0022022117713153

[B2] Adelmann P. K. (1995). Emotional labor as a potential source of job stress, in Organizational Risk Factors for Job Stress, eds Sauter S. L. Murphy L. R. (Washington, DC: American Psychological Association), 371–381. 10.1037/10173-023

[B3] Allen J. A. Diefendorff J. M. Ma Y. (2014). Differences in emotional labor across cultures: a comparison of Chinese and US service workers. J. Bus. Psychol. 29, 21–35. 10.1007/s10869-013-9288-7

[B4] Ashforth B. E. Humphrey R. H. (1993). Emotional labor in service roles: the influence of identity. Acad. Manage. Rev. 18, 88–115. 10.5465/amr.1993.3997508

[B5] Bakker A. B. Heuven E. (2006). Emotional dissonance, burnout, and in-role performance among nurses and police officers. Int. J. Stress Manage. 13, 423–440. 10.1037/1072-5245.13.4.423

[B6] Bansal P. Corley K. (2011). The coming of age for qualitative research: embracing the diversity of qualitative methods. Acad. Manage. J. 54, 233–237. 10.5465/amj.2011.60262792

[B7] Brislin R. W. (1980). Translation and content analysis of oral and written material, in Handbook of Cross-Cultural Psychology: Methodology, eds Triandis H. C. Berry J. W. (Boston: Allyn and Bacon, 389–444.

[B8] Brotheridge C. M. Grandey A. A. (2002). Emotional labor and burnout: comparing two perspectives of “people work. J. Vocat. Behav. 60, 17–39. 10.1006/jvbe.2001.1815

[B9] Brotheridge C. M. Lee R. T. (2003). Development and validation of the emotional labour scale. J. Occup. Organ. Psych. 76, 365–379. 10.1348/096317903769647229

[B10] Cammann C. Fichman M. Jenkins D. Klesh J. (1979). The Michigan Organizational Assessment Questionnaire. Unpublished manuscript. University of Michigan, Ann Arbor, MI.

[B11] Chen K. Y. Chang C. W. Wang C. H. (2019). Frontline employees' passion and emotional exhaustion: the mediating role of emotional labor strategies. Int. J. Hosp. Manag. 76, 163–172. 10.1016/j.ijhm.2018.05.006

[B12] Chen Z. Sun H. Lam W. Hu Q. Huo Y. Zhong J. A. (2012). Chinese hotel employees in the smiling masks: roles of job satisfaction, burnout, and supervisory support in relationships between emotional labor and performance. Int. J. Hum. Resour. Man. 23, 826–845. 10.1080/09585192.2011.579918

[B13] Cheng J.-C. Yang O.-Y. (2018). Hotel employee job crafting, burnout, and satisfaction: the moderating role of perceived organizational support. Int. J. Hosp. Manag. 72, 78–85. 10.1016/j.ijhm.2018.01.005

[B14] Cheung F. Tang C. S.-,k. Tang S. (2011). Psychological capital as a moderator between emotional labor, burnout, and job satisfaction among school teachers in China. Int. J. Stress Manage. 18, 348–371. 10.1037/a0025787

[B15] Cheung F. Y. Tang C. S. K. (2010). Effects of age, gender, and emotional labor strategies on job outcomes: moderated mediation analyses. Appl. Psychol. Health Well Being. 2, 323–339. 10.1111/j.1758-0854.2010.01037.x

[B16] Cheung F. Y.-L. Tang C. S.-K. (2009). Quality of work life as a mediator between emotional labor and work family interference. J. Bus. Psychol. 24, 245–255. 10.1007/s10869-009-9103-7

[B17] Chi N. W. Grandey A. A. (2019). Emotional labor predicts service performance depending on activation and inhibition regulatory fit. J. Manag. 45, 673–700. 10.1177/0149206316672530

[B18] Chi S.-C. S. Friedman R. A. Chu C.-C. Shih H.-L. (2019). Chinese acceptance of mistreatment by in-relation offenders can be neutralized by triggering a “group” collectivism perspective. Eur. J. Work Organ. Psychol. 28, 384–398. 10.1080/1359432X.2019.1585809

[B19] Choi H. Oishi S. Shin J. Suh E. M. (2019). Do happy events love company? Cultural variations in sharing positive events with others. Pers. Soc. Psychol. Bull. 45, 528–540. 10.1177/0146167218789071 30141381

[B20] Choi H.-M. Mohammad A. A. A. Kim W. G. (2019). Understanding hotel frontline employees' emotional intelligence, emotional labor, job stress, coping strategies and burnout. Int. J. Hosp. Manag. 82, 199–208. 10.1016/j.ijhm.2019.05.002

[B21] Chu K. H.-L. Murrmann S. K. (2006). Development and validation of the hospitality emotional labor scale. Tourism Manage. 27, 1181–1191. 10.1016/j.tourman.2005.12.011

[B22] Churchill G. A. (1979). A paradigm for developing better measures of marketing constructs. J. Mark. Res. 16, 64–73. 10.1177/002224377901600110

[B23] Cossette M. Hess U. (2015). Service with style and smile. How and why employees are performing emotional labour? Eur. Rev. Appl. Psychol. 65, 71–82. 10.1016/j.erap.2015.02.001

[B24] Creswell J. W. Hanson W. E. Clark Plano V. L. Morales A. (2007). Qualitative research designs: selection and implementation. Couns. Psychol. 35, 236–264. 10.1177/0011000006287390

[B25] Devitt P. (2003). Qualitative research and evaluation methods (third edition). Nurs. Educ. Today. 23, 467–467. 10.1016/S0260-6917(03)00079-0

[B26] Diefendorff J. M. Croyle M. H. Gosserand R. H. (2005). The dimensionality and antecedents of emotional labor strategies. J. Vocat. Behav. 66, 339–357. 10.1016/j.jvb.2004.02.001

[B27] Diefendorff J. M. Richard E. M. (2003). Antecedents and consequences of emotional display rule perceptions. J. Appl. Psychol. 88, 284–294. 10.1037/0021-9010.88.2.284 12731712

[B28] Eid M. Diener E. (2001). Norms for experiencing emotions in different cultures: inter- and intranational differences. J. Pers. Soc. Psychol. 81, 869–885. 10.1037/0022-3514.81.5.869 11708563

[B29] Ekman P. Friesen W. V. (1969). The repertoire of nonverbal behavior: categories, origins, usage, and coding. Semiotica. 1, 49–98. 10.1515/semi.1969.1.1.49

[B30] Erickson R. J. Wharton A. S. (1997). Inauthenticity and depression: assessing the consequences of interactive service work. Work Occup. 24, 188–213. 10.1177/0730888497024002004

[B31] Farh J.-L. Cannella A. A. Lee C. (2006). Approaches to scale development in Chinese management research. Manag. Organ. Rev. 2, 301–318. 10.1111/j.1740-8784.2006.00055.x

[B32] Gabriel A. S. Daniels M. A. Diefendorff J. M. Greguras G. J. (2015). Emotional labor actors: a latent profile analysis of emotional labor strategies. J. Appl. Psychol. 100, 863–879. 10.1037/a0037408 25068812

[B33] Glomb T. M. Tews M. J. (2004). Emotional labor: a conceptualization and scale development. J. Vocat. Behav. 64, 1–23. 10.1016/S0001-8791(03)00038-1

[B34] Goffman E. (1959). The moral career of the mental patient. Psychiatry 22, 123–142. 10.1080/00332747.1959.11023166 13658281

[B35] Grandey A. A. (2000). Emotional regulation in the workplace: a new way to conceptualize emotional labor. J. Occup. Health Psychol. 5, 95–110. 10.1037/1076-8998.5.1.95 10658889

[B36] Grandey A. A. (2003). When “the show must go on”: surface acting and deep acting as determinants of emotional exhaustion and peer-rated service delivery. Acad. Manage. J. 46, 86–96. 10.5465/30040678

[B37] Grandey A. A. Fisk G. M. Mattila A. S. Jansen K. J. Sideman L. A. (2005). Is “service with a smile” enough? Authenticity of positive displays during service encounters. Organ. Behav. Hum. Decis. Process. 96, 38–55. 10.1016/j.obhdp.2004.08.002

[B38] Grandey A. A. Frone M. R. Melloy R. C. Sayre G. M. (2019). When are fakers also drinkers? A self-control view of emotional labor and alcohol consumption among US service workers. J. Occup. Health Psychol. 24, 482–497. 10.1037/ocp0000147 30829513 PMC6675660

[B39] Groth M. Hennig-Thurau T. Walsh G. (2009). Customer reactions to emotional labor: the roles of employee acting strategies and customer detection accuracy. Acad. Manage. J. 52, 958–974. 10.5465/amj.2009.44634116

[B40] Hennig-Thurau T. Groth M. Paul M. Gremler D. D. (2006). Are all smiles created equal? How emotional contagion and emotional labor affect service relationships. J. Mark. 70, 58–73. 10.1509/jmkg.70.3.58

[B41] Hinkin T. R. (1995). A review of scale development practices in the study of organizations. J. Manag. 21, 967–988. 10.1177/014920639502100509

[B42] Hochschild A. R. (1979). Emotion work, feeling rules, and social structure. Am. J. Sociol. 85, 551–575. 10.1086/227049

[B43] Hochschild A. R. (1983). The Managed Heart: Commercialization of Human Feeling. Berkeley, CA: University of California Press.

[B44] Hofstede G. (1980). Motivation, leadership, and organization: do American theories apply abroad? Organ. Dyn. 9, 42–63. 10.1016/0090-2616(80)90013-3

[B45] Humphrey R. H. Ashforth B. E. Diefendorff J. M. (2015). The bright side of emotional labor. J. Organ. Behav. 36, 749–769. 10.1002/job.2019

[B46] Johnson R. E. Muraven M. Donaldson T. L. Lin S.-H. (2018). Self-control in work organizations, in The Self at Work: Fundamental Theory and Research, eds Ferris D. L. Johnson R. E. Sedikides C. (New York, NY: Routledge/Taylor & Francis Group, 119–144.

[B47] Jordan C. Soutar G. Kiffin-Petersen S. (2008). Are there different “types” of emotional laborers?, in Paper presented at the Sixth International Conference on Emotions and Organizational Life (Fontainebleau: EMONET VI).

[B48] Judge T. A. Woolf E. F. Hurst C. (2009). Is emotional labor more difficult for some than for others? A multilevel, experience-sampling study. Pers. Psychol. 62, 57–88. 10.1111/j.1744-6570.2008.01129.x

[B49] Kinman G. Wray S. Strange C. (2011). Emotional labour, burnout and job satisfaction in UK teachers: the role of workplace social support. Educ. Psychol. 31, 843–856. 10.1080/01443410.2011.608650

[B50] Kitirattarkarn G. P. Araujo T. Neijens P. (2019). Challenging traditional culture? How personal and national collectivism-individualism moderates the effects of content characteristics and social relationships on consumer engagement with brand-related user-generated content. J. Advert. 48, 197–214. 10.1080/00913367.2019.1590884

[B51] Korman A. K. (1974). Contingency approaches to leadership: an overview, in Contingency Approaches to Leadership, eds Hunt J. G. Larson L. L. (Carbondale, IL: Southern Illinois University Press, 189–195.

[B52] Kruml S. M. Geddes D. (2000). Exploring the dimensions of emotional labor: the heart of Hochschild's work. Manag. Commun. Q. 14, 8–49. 10.1177/0893318900141002

[B53] Lawler E. E. III. (1973). Motivation in Work Organization. Monterey. CA: Brooks/Cole.

[B54] Liu C. Luo Y. (2019). Cross-cultural examination of Chinese cross-border e-commerce web sites design and contents. in 2019 International Conference on Management, Education Technology and Economics (ICMETE 2019) (Fuzhou). 10.2991/icmete-19.2019.106

[B55] Lu X. Guy M. E. (2019). Emotional labor, performance goal orientation, and burnout from the perspective of conservation of resources: a United States/China comparison. Public Perform. Manag. Rev. 42, 685–706. 10.1080/15309576.2018.1507916

[B56] Luo A. Guchait P. Lee L. Madera J. M. (2019). Transformational leadership and service recovery performance: the mediating effect of emotional labor and the influence of culture. Int. J. Hosp. Manag. 77, 31–39. 10.1016/j.ijhm.2018.06.011

[B57] Maslach C. Jackson S. E. Leiter M. P. Schaufeli W. B. Schwab R. L. (1986). Maslach Burnout Inventory. Palo Alto, CA: Consulting Psychologists Press.

[B58] Maslach C. Schaufeli W. B. Leiter M. P. (2001). Job burnout. Annu. Rev. Psychol. 52, 397–422. 10.1146/annurev.psych.52.1.397 11148311

[B59] Masuda T. Ellsworth P. C. Batja M. Janxin L. Shigehito T. Ellen V. D. V. (2008). Placing the face in context: cultural differences in the perception of facial emotion. J. Pers. Soc. Psychol. 94, 365–381. 10.1037/0022-3514.94.3.365 18284287

[B60] Matsumoto D. (2007). Individual and cultural differences on status differentiation: the status differentiation scale. J. Cross-Cult. Psychol. 38, 413–431. 10.1177/0022022107302311

[B61] Mishra S. K. Bhatnagar D. D'Cruz P. Noronha E. (2012). Linkage between perceived external prestige and emotional labor: mediation effect of organizational identification among pharmaceutical representatives in India. J. World Bus. 47, 204–212. 10.1016/j.jwb.2011.04.007

[B62] Morris J. A. Feldman D. C. (1996). The dimensions, antecedents, and consequences of emotional labor. Acad. Manage. Rev. 21, 986–1010. 10.5465/amr.1996.9704071861

[B63] Nixon A. E. Ceylan S. Nelson C. E. Alabak M. (2019). Emotional labour, collectivism and strain: a comparison of Turkish and US service employees. Work Stress. 10.1080/02678373.2019.1598515. [Epub ahead of print].

[B64] Noon J. M. Lewis J. R. (1992). Therapeutic strategies and outcomes: perspectives from different cultures. Brit. J. Med. Psychol. 65, 107–117. 10.1111/j.2044-8341.1992.tb01691.x 1633116

[B65] Podsakoff P. M. Organ D. W. (1986). Self-reports in organizational research: problems and prospects. J. Manag. 12, 531–544. 10.1177/014920638601200408

[B66] Pugh S. D. Groth M. Hennig-Thurau T. (2011). Willing and able to fake emotions: a closer examination of the link between emotional dissonance and employee well-being. J. Appl. Psychol. 96, 377–390. 10.1037/a0021395 21058805

[B67] Pugliesi K. (1999). The consequences of emotional labor: effects on work stress, job satisfaction, and well-being. Motiv. Emotion. 23, 125–154. 10.1023/A:1021329112679

[B68] Rutter D. R. Fielding P. J. (1988). Sources of occupational stress: an examination of British prison officers. Work Stress. 2, 291–299. 10.1080/02678378808257490

[B69] Safdar S. Friedlmeier W. Matsumoto D. Yoo S. H. Kwantes C. T. Kakai H. (2009). Variations of emotional display rules within and across cultures: a comparison between Canada, USA, and Japan. Can. J. Behav. Sci. 41, 1–10. 10.1037/a0014387

[B70] Scherr S. Mares M.-L. Bartsch A. Goetz M. (2019). Parents, television, and children's emotional expressions: a cross-cultural multilevel model. J. Cross-Cult. Psychol. 50, 22–46. 10.1177/0022022118806585

[B71] Teng H.-Y. (2019). Job crafting and customer service behaviors in the hospitality industry: mediating effect of job passion. Int. J. Hosp. Manag. 81, 34–42. 10.1016/j.ijhm.2019.03.013

[B72] Trougakos J. P. Beal D. J. Cheng B. H. Hideg I. Zweig D. (2015). Too drained to help: a resource depletion perspective on daily interpersonal citizenship behaviors. J. Appl. Psychol. 100, 227–236. 10.1037/a0038082 25314365

[B73] Uchida Y. Kitayama S. Mesquita B. Reyes J. A. S. Morling B. (2008). Is perceived emotional support beneficial? Well-being and health in independent and interdependent cultures. Pers. Soc. Psychol. Bull. 34, 741–754. 10.1177/0146167208315157 18359927

[B74] van Hemert D. A. Poortinga Y. H. van de Vijver F. J. R. (2007). Emotion and culture: a meta-analysis. Cogn. Emot. 21, 913–943. 10.1080/02699930701339293

[B75] Wang X. P. (2018). National Bureau of Statistics of China: Service Industry has Become the Largest Industry in the National Economy. Available online at: http://finance.youth.cn/finance_gdxw/201804/t20180415_11598851.htm (accessed April 15, 2018).

[B76] Wen J. Huang S. Hou P. (2019). Emotional intelligence, emotional labor, perceived organizational support, and job satisfaction: a moderated mediation model. Int. J. Hosp. Manag. 81, 120–130. 10.1016/j.ijhm.2019.01.009

[B77] Yanchus N. J. Eby L. T. Lance C. E. Drollinger S. (2010). The impact of emotional labor on work–family outcomes. J. Vocat. Behav. 76, 105–117. 10.1016/j.jvb.2009.05.001

[B78] Yang Y. Hong Y.-,y. Sanchez-Burks J. (2019). Emotional aperture across east and west: how culture shapes the perception of collective affect. J. Cross-Cult. Psychol. 50, 751–762. 10.1177/0022022119846412

[B79] Yin H. (2012). Adaptation and validation of the teacher emotional labour strategy scale in China. Educ. Psychol. 32, 451–465. 10.1080/01443410.2012.674488

[B80] Yin H. Huang S. Chen G. (2019). The relationships between teachers' emotional labor and their burnout and satisfaction: a meta-analytic review. Educ. Res. Rev. 28:100283 10.1016/j.edurev.2019.100283

[B81] Zapf D. (2002). Emotion work and psychological well-being: a review of the literature and some conceptual considerations. Hum. Resour. Manage. R. 12, 237–268. 10.1016/S1053-4822(02)00048-7

[B82] Zhang Q. Zhu W. (2008). Exploring emotion in teaching: emotional labor, burnout, and satisfaction in Chinese higher education. Commun. Educ. 57, 105–122. 10.1080/03634520701586310

